# Chasing More Than Opioids: Gambling Harm in Patients Receiving Opioid Substitution Therapy (OST)

**DOI:** 10.1192/bjo.2026.11205

**Published:** 2026-06-30

**Authors:** Mohammad Ali

**Affiliations:** Addictions Recovery Community (ARC-MK), Milton Keynes, United Kingdom

## Abstract

**Aims:**

Gambling-related harm is under-recognised in patients receiving opioid substitution treatment (OST). This study aimed to determine the prevalence and severity of gambling harm in an OST outpatient population, explore associations with concurrent substance use (cocaine, cannabis, illicit benzodiazepines), and assess the feasibility of routine screening in clinical practice.

**Methods:**

A cross-sectional service evaluation was conducted in an NHS addictions psychiatry outpatient service. Adults aged ≥18 years receiving methadone or buprenorphine were opportunistically screened during routine appointments, excluding those unable to engage due to acute intoxication, severe withdrawal, cognitive impairment, or acute psychiatric instability. Gambling-related harm was assessed using the Problem Gambling Severity Index (PGSI). Demographic data, opioid substitution treatment type, concurrent substance use, and psychiatric comorbidities were extracted from clinical records and patient self-report. Data were anonymised and analysed descriptively, with exploratory comparisons between patients with and without gambling-related harm.

**Results:**

A total of 62 patients were screened. Eighteen patients (29%) scored ≥3 on the Problem Gambling Severity Index (PGSI), indicating moderate-risk or problem gambling, while nine patients (14%) met criteria for problem gambling (PGSI ≥8). Gambling-related harm was more frequently observed among patients reporting concurrent cocaine use and illicit benzodiazepine use. The majority (82%) of patients with moderate or severe gambling harm had no prior documentation of gambling difficulties in their clinical records, suggesting significant under-recognition. Screening was feasible, added minimal time to routine assessments, and prompted clinically relevant discussions around financial stress and relapse risk.

**Conclusion:**

Gambling-related harm appears common yet under-identified among patients receiving OST in addiction psychiatry outpatient services. Routine screening using brief validated tools such as the PGSI is feasible and may enhance holistic assessment and care planning. Integrating gambling harm assessment into standard addiction reviews may help address unmet clinical need and improve outcomes in this complex patient group.

